# Implementation Outcomes and Recommendations of Two Physical Activity Interventions: Results from the Danish ACTIVE SCHOOL Feasibility Study

**DOI:** 10.3390/ijerph22010067

**Published:** 2025-01-07

**Authors:** Lise Sohl Jeppesen, Jesper Sandfeld, Søren Smedegaard, Glen Nielsen, Mathias Brekke Mandelid, Malene Norup, Jacob Wienecke, Anna Bugge

**Affiliations:** 1 Department of Nutrition, Exercise and Sports, University of Copenhagen, DK-2200 Copenhagen, Denmark; gnielsen@nexs.ku.dk (G.N.); wienecke@nexs.ku.dk (J.W.); 2 Department of Midwifery, Physiotherapy, Occupational Therapy and Psychomotor Therapy, University College Copenhagen, DK-2200 Copenhagen, Denmark; mals@kp.dk (M.N.); abug@kp.dk (A.B.); 3 Faculty of Teacher Education, University College Copenhagen, DK-1799 Copenhagen, Denmark; jesm@kp.dk; 4 Faculty of Education and Social Sciences, UCL University College, DK-5230 Odense, Denmark; sosm@ucl.dk; 5 Department of Pedagogy, Social Science and Religion, Faculty of Education, Arts and Sports, Western Norway University of Applied Sciences, NO-6856 Sogndal, Norway; mathias.brekke.mandelid@hvl.no; 6 Department of Sports, Physical Education and Outdoor Studies, Faculty of Humanities, Sports and Educational Science, University of South-Eastern Norway, NO-3800 Bø, Norway

**Keywords:** primary school, children, teachers, learning, health interventions

## Abstract

Physical activity (PA) should be an essential part of all children’s lives, as it can promote physical and mental health, enhance general well-being, and positively impact learning outcomes. Schools offer an ideal setting to encourage physical activity during the school day, as nearly all children attend school. However, schools present a complex environment for implementing PA, and sedentary behavior is common in classroom teaching. This study explores the feasibility of two types of school-based physical activity interventions: one based on research in exercise and cognition (Run, Jump & Fun) and another grounded in embodied learning (Move & Learn). Run, Jump & Fun can be conceptualized as extra non-curricular physical activity implemented into the school day while Move & Learn is integrated into curricular time. The 8-week study involved third-grade students and their teachers from seven schools, with educational strategies applied to support adoption. Quantitative and qualitative data were collected before, during, and after the 8-week period. The results indicated that both interventions were feasible to implement, leading to a series of recommendations for further refinement. These recommendations can guide the development of future school-based PA interventions and inspire other researchers to assess and improve their implementation strategies.

## 1. Introduction

Physical activity (PA) should be an essential part of all children’s lives as it can promote physical and mental health [1,2], enhance positive social aspects [3], increase motivation for school and general well-being [4], and positively affect learning outcomes [5,6,7]. For these reasons, research has advocated schools as an advantageous setting for PA promotion, as almost all children attend school, including those with different levels of physical prerequisites and abilities, and children from different social backgrounds [8,9,10]. However, the school is a very complex setting [11] and sedentary learning activities are predominant during the whole school day, for example, in classroom teaching [12,13,14].

There has been increasing interest in exploring how implementing different types of school-based PA interventions can improve children’s physical and mental health [15]. PA in school is a multifaceted and broad phenomenon, as it can be applied in multiple ways. This breadth is illustrated by the many types and concepts of PA in schools [16,17]. Moderate-to-Vigorous PA (MVPA) integrated into the school day has been demonstrated to induce physiological adaptations in the brain [6,16,17,18,19], have a positive effect on cognitive outcomes [20,21] and increase academic performance [5,15,20,21]. Another concept is Embodied Learning (EL) [22]. It comprises task-relevant bodily engagement in learning activities [23]. Embodied learning has shown positive associations with enhanced academic performance [22,23], particularly in language and mathematics [24,25]. Furthermore, some studies have found that understandings of abstract concepts can be increased by enactive engagement with the subject matter [26,27]. Embodied learning is primarily performed with a low intensity, but with bodily engagement in tasks relevant to the curriculum, such as math [22]. Accordingly, both PA with a focus on moderate-to-vigorous intensity, physically active learning activities, and specific embodied learning sessions have been shown to potentially enhance academic performance in schools.

The school setting is complex and has several systems and levels affecting each other, including pupils, staff, management, and policy [28]. Therefore, methods and concepts which can translate PA and embodied learning into meaningful and feasible teaching didactics for this complex setting are needed [29]. Further research is still needed, e.g., in terms of assessing factors with importance to the adoption and implementation of PA interventions implemented by teachers [30], and fidelity to the prescribed PA intensity and duration of school-based interventions [31]. Overall, there is a need for research on the feasibility of ways to enhance and implement PA in the school setting [30,32,33,34,35,36].

Teachers and school pedagogues (grouped and hereafter called “teachers” in this paper) delivered the interventions in this feasibility study. The novelty of this study lies in several aspects. Studies with PA interventions designed in collaboration with and delivered by teachers are still not common, even though this approach has been suggested to enhance sustainability [37]. Furthermore, the study’s two interventions aim to adhere tightly to the evidence of embodied learning and additional MVPA to develop feasible and specific interventions that do not mix multiple theoretical foundations, thereby ensuring a valid base for explaining any potential effects. Other studies in the field have used a mixture of evidence in designing their interventions, and it is therefore difficult to single out the effects of different elements of the interventions [38,39]. Finally, both interventions align with the Danish school’s education purpose, as they are designed to support the school’s learning objectives and pedagogical goals [40].

In response to the challenges of translating and conceptualizing PA interventions and innovations into teachers’ and schools’ practice, the research project ACTIVE SCHOOL led a design process which preceded the development of two different PA interventions: (1) “Run, Jump & Fun” (RJF) and (2) “Move & Learn” (ML) [41]. The current study’s objective is to assess the feasibility and implementation of these two interventions; RJF based on research regarding exercise and cognition and ML based on research regarding embodied learning.

This feasibility study aims to inform the development of other teacher-led school-based PA interventions with its recommendations, and to inspire other researchers of school-based PA to evaluate their implementation strategies. The study’s recommendations are outlined in the closing part of the discussion. We hypothesized that the two interventions would be both feasible and implementable according to the teachers.

## 2. Materials and Methods

### 2.1. Study Design

This study investigates the feasibility and implementation of the two types of school-based PA with teachers as the implementers. This was assessed in terms of *Acceptability* (how the interventions are accepted to be implemented by the teachers), *Feasibility* (whether the teachers found the interventions possible to implement in the school context or not), *Appropriateness* (if the interventions are regarded as relevant, profitable and implementable by the teachers), *Adoption* (how teachers adopt the intervention instructions in their practice), and *Fidelity* (whether teachers adhere to the interventions as intended). The study was based on a pre-post quasi-experimental intervention for two groups of teachers and their pupils in the third grade from seven different schools (non-randomized). The study focused on the feasibility of the two interventions, and not on whether one of them showed the most promising outcomes. Therefore, we did not compare the results from the two intervention groups. The system climate and support from school leaders, colleagues, and parents, as factors affecting feasibility, were explored in interviews with the teachers after the intervention period. Multiple quantitative and qualitative methods assessed feasibility (see overview in Table 1). 

This study used a mixed methods convergent basic design comprising integration at the theory, design, method, and interpretation levels [42,43]. The Consolidated Framework for Implementation Research Outcomes Addendum Diagram (CFIR OAD) served as a theoretical frame for the data collection, analyses, and synthesis [44]. The inquiries were applied to the same population and setting in the same study period. The qualitative and quantitative data analyses were merged and synthesized to provide multiple perspectives and explain the implementation findings from the two interventions in depth.

The study had a recruitment phase from April to June 2022. The recruited schools were introduced and prepared for the study by the research group from mid-August to the end of September 2022. The interventions lasted 8 weeks from the end of September to the end of November 2022. Survey data on appropriateness and feasibility were obtained at two time points, week 0 (baseline) and week 8 (post-intervention), and interviews with teachers were performed post-intervention, while fidelity data (adoption and adherence to target minutes) were collected once a week during the 8 weeks.

### 2.2. Schools and Participants

Schools were recruited for this feasibility study through networking and advertising on Facebook and in the largest Danish School magazine both online and in printed volumes.

Eligible schools for the study were both private and public primary schools in Denmark. Schools with an interest in participation received information material, and a meeting was held to ensure sufficient information was given and that the school’s management team was motivated and would prioritize personnel for the project before signing the school participation form. Seven schools from three regions of Denmark were included. Furthermore, all teachers in the included 3rd grade classes were eligible to participate. Participating teachers signed a written consent form agreeing to participate in the delivery of the interventions and in the scientific exploration of feasibility.

Schools could choose between the two interventions: RJF and ML. Three schools signed up for RJF, and four schools signed up for ML. In total, 32 school staff members (RJF, *n* = 15 and ML, *n* = 17) were enrolled in the study. Of these, 23 school staff members participated in surveys and group interviews (RJF *n* = 11 and ML *n* = 12). The teachers’ main teaching subjects were math or Danish. This is outlined below under “Procedures and measures”.

### 2.3. The Interventions

Prior to this study, the two interventions, RJF and ML, were designed in a one-year educational design study involving school stakeholders and experts [41]. Both interventions consisted of specific intervention characteristics and concomitant implementation strategies, as shown in Table 2.

Education of deliverers (teachers) is a common strategy in implementation science, as it builds up capacity within the organization responsible for implementing the intervention [45]. Therefore, for each intervention, two courses were held for teachers focusing on exemplary activities, planning tools, and didactics. The courses were delivered by staff from the University College Copenhagen teacher and pedagogue education departments, who were experienced in delivering continuing professional development to teachers. Additionally, both interventions included team meetings as a strategy carried out by the local teachers to create an environment for the adoption and implementation of the interventions at each school. The ACTIVE SCHOOL team provided meeting agendas and intervention-specific process themes for these meetings. The school leader was encouraged to lead the meetings. An overview of the interventions and their accompanying strategies is shown in Table 1, which is followed by a detailed description of distinctive intervention characteristics.

#### 2.3.1. Run, Jump & Fun Intervention 

Staff delivery: The dose of 4 × 30 min of RJF activity distributed on four days pr. week was based on earlier studies implementing similar doses of PA, e.g., a Norwegian study reporting positive outcomes from interventions where students had 120 min of extra PA per week added to their mandatory PE [46]. Further, the majority of intervention studies in a recent review by Singh and colleagues had a dose of at least 30 min extra PA per session, and many of these for five days a week [5].

Staff capacity: The courses included the core elements of RJF activities, such as keeping the intensity moderate to vigorous, but also focused on attributes like motivation, inclusion, and fostering mastery among all pupils. RJF principles guided this practice. This encouraged teachers to adhere to MVPA intensity and set up pedagogical goals for the activities as well. Examples of activities included diverse ball games, agility and obstacle courses, and balloon badminton. The course content for RJF consisted of an introduction to a tailored planning model for activities, practicing exemplary games and exercises with the principles of the intervention. Furthermore, materials with exemplary lesson activities and a planning model were provided.

System climate: The establishment process was unique for RJF and was a result of the design study that preceded this study [41]. The process aimed to generate shared ownership between the organizational level, the management of the school, and the practice level, the teachers. This process consisted of four meetings, starting six weeks before the intervention began and continuing throughout the intervention period. The intervention was coordinated at these meetings, and a mutual understanding of the intervention was established between the school principal and the teachers. Furthermore, teachers were encouraged to conduct team meetings during the intervention period with RJF on the agenda. ACTIVE SCHOOL provided guidance for the meetings. See Table 1 for an overview.

#### 2.3.2. Move & Learn Intervention

Staff delivery: The dose of ML of 4 × 30 min of embodied learning distributed in two Danish and two math lessons pr. week was based on other studies showing positive results after implementing a similar dose [5,46]. The focus during the ML intervention was on delivering embodied learning activities, where the body and its movements are integrated into the learning session with curricular content. Examples of ML activities used for teaching Danish could be pupils miming animals from fables they had written themselves. Examples of ML activities used for teaching math could be learning about displacements in large coordinate grids in the schoolyard, where the body moves around as x and y in the coordinates.

Staff capacity: Through courses, the Danish and math teachers were introduced to embodied learning theory and practice. These courses allowed for mutual discussions and feedback from the course educator. A didactic model with different types of bodily action was the focal point for the teachers when planning their own teaching using ML. A set of five intervention principles (see Supplementary Materials I) guided the ML intervention. The principles encouraged the teachers to adopt embodied learning as a teaching method with specific instructions. The teachers’ own progression and use of ML principles were particularly emphasized in the second ML course.

Furthermore, materials with exemplary lesson activities, QR-codes displaying teaching videos, and a didactic model were provided.

System climate: For ML no establishment process was included as a strategy, because the ML intervention was implemented in ordinary Danish and math lessons, and thus, did not require additional time outside the ordinary schedule. Teachers were encouraged to conduct team meetings in the intervention period with ML on the agenda. Guidance for the meetings was provided by ACTIVE SCHOOL. See Table 1 for an overview.

### 2.4. Procedures and Measures

The selection of relevant implementation outcomes was guided by the Consolidated Framework of Implementation Research Outcomes Addendum Diagram (CFIR OAD) [44], and these outcomes explored through several inquiries, as shown in Table 1.

The procedures and measures will be presented in the following sections: Quantitative Measures, Statistics, Qualitative Data, and Analysis. 

#### 2.4.1. Quantitative Measures

A validated survey Usage Rating Profile-Intervention (URP-I) [47] was used to assess implementation. The administration and scoring procedures of the questionnaire followed the prescribed procedures [47]. The original survey was translated into Danish using the World Health Organization (WHO) procedure for translating measure tools within health research [48]. The survey was administered to school staff implementing the interventions. It was sent out twice, once before the intervention period and once after the 8-week intervention. The survey was adapted to be specific to ACTIVE SCHOOL activities and consisted of 24 items, and only items relevant to this project were included. All items were scored from 1 = strongly disagree to 6 = strongly agree. Sum scores were calculated for each factor. The complete questionnaire is presented in Supplementary Materials III in this paper. Further intervention-specific post-implementation questions were added to the second URP-I survey to evaluate *appropriateness*, *adherence*, *adoption*, *implementation* and *sustainability* and were scored separately. This had a standardized administration and scoring procedures identical to that of the URP-I. However, the extent of the survey was not validated across larger populations.

To measure the *fidelity* of the interventions, a short survey was sent out using the Survey Xact system in an SMS every Friday during the eight weeks. The questions covered adherence to the intervention principles for ML and RJF (see Supplementary Materials IV). Furthermore, teachers had to report how many minutes they delivered their intervention each day during the week. This survey was standardized in its administration and scoring procedures.

#### 2.4.2. Statistics

Data from the URP-I survey and the weekly SMS questions about adherence are presented using descriptive statistics. The quantitative data are presented as case-study data specific to the study’s context, with unknown generalizability, as the sample size is too low.

#### 2.4.3. Qualitative Data

Seven group interviews were conducted after the intervention period to obtain a deeper understanding of the implementation. In total, 23 informants participated in the interviews (teachers *n* = 19, pedagogues *n* = 3, school principal *n* = 1). The first author conducted the interviews. The interviews had an interview guide grounded in CFIR OAD (see interview guide in Supplementary Materials IV). The focus group interviews were transcribed verbatim and were coded in Nvivo 14 (Release 1.7.1) by Lumivero, Denver, Colorado. The coding followed the concept of reflexive thematic analysis [49].

#### 2.4.4. Analysis of Qualitative Data

The analysis was divided into six phases: (1) familiarizing, (2) coding, (3) developing initial themes, (4) developing and reviewing themes, (5) refining, defining, and naming themes, and (6) writing up [49].

The “familiarizing” only focused on overarching concepts, for example, “adoption”. The “coding” phase had a free and inductive approach to exploring semantic and latent meanings. The analysis became deductive, as the process progressed to “initial themes” and “developing and reviewing themes”. When “refining, defining, and naming themes” the researcher consolidated the analysis’s unique terminology for themes related to this project. However, the researcher constructed the themes to uncover, for example, “understanding” and how it was conceived in this study. In the “writing up” phase, perspectives associated with findings from the quantitative data sources were merged into the analysis.

## 3. Results

First, *acceptability*, *understanding*, *feasibility* and *appropriateness*, which were assessed as antecedents of implementation, are presented, followed by the implementation outcomes, *adoption* and *fidelity*. The results of each intervention (RJF and ML) are presented separately, starting each time with quantitative data displaying the degree of the implementation outcomes. This is followed by a presentation of the qualitative data, demonstrating how the teachers implemented the interventions.

### 3.1. Acceptability, Understanding, and Feasibility

#### 3.1.1. Run, Jump & Fun

**Figure 1:**
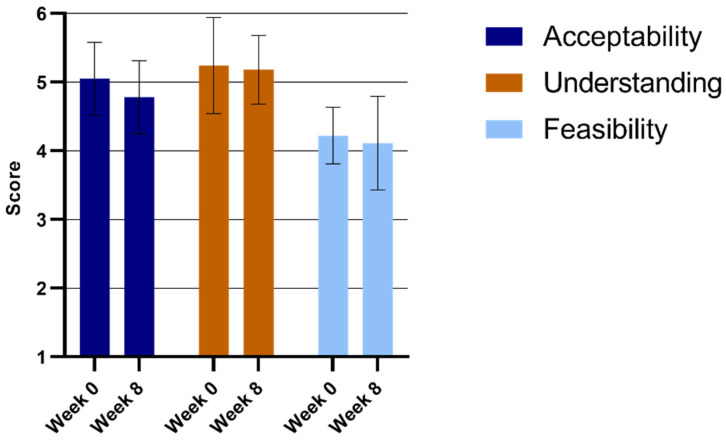
RJF deliverers’ Usage Rating Profile-Intervention (URP-I) subscale scores.

Participants reported high levels of RJF intervention acceptability and understanding in week 0 and week 8 (Figure 1). This indicates that teachers went from having a theoretical and assumed understanding to an equally rated applied practical understanding of the interventions after eight weeks. The feasibility scores for RJF remained unchanged and relatively high from week 0 (Mean = 4.22, sd = 0.41) to week 8 (Mean = 4.11, sd = 0.68), indicating that the intervention was feasible.

In the qualitative data regarding the RJF intervention, some teachers reported that they did not have enough time to implement the intervention. It was difficult for them to prioritize 30 min of MVPA four times a week within their regular class schedule, alongside the extra time required for preparing activities and conducting meetings related to the intervention.

“*…it takes a long time to do, I think. Both in terms of preparation and execution. Maybe longer than I thought*”

(School 3, Teacher 2)

The courses, however, helped teachers to save time, since they enabled them to improvise:

“*What we did (in the courses, ed.) was very concrete. It was something we could take home and bring into the classroom right away or just use on the playground. So, it was a good bank of ideas to take home, if you were completely blank. I mean, before you went on the course…*”

(School 5, Teacher 2)

The overall thematic reflexive analysis of the qualitative data for the RJF intervention led to the construction of two core themes, *boarding to start building* and *hands-on engagement matters*, covering the acceptability of the RJF intervention (see the theme table in Supplementary Materials Section V).

The theme *boarding to start building* refers to the thorough onboarding process, which led to the building of a local school model and the teachers’ translation of RJF. Many of the RJF teachers were PE teachers with values which aligned with a positive outlook regarding pupils’ exercise and movement.

The core theme *hands-on engagement matters* unfolded the feasibility of the intervention further. This theme was inseparably linked to the individual teaching styles of the teachers. Numerous thematic codes showed that the implementation strategy elements of the theories and written material did not have a significant impact on feasibility for this group. Instead, the feasibility arose from bodily experiences during the intervention courses.

#### 3.1.2. Move & Learn

**Figure 2:**
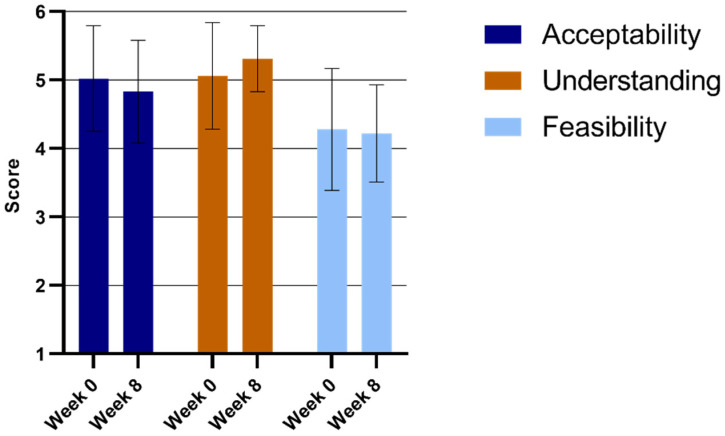
ML deliverers’ Usage Rating Profile-Intervention (URP-I) subscale scores.

The teachers implementing the ML intervention reported high levels of acceptability from week 0 (mean = 5.02; SD = 0.77) to week 8 (mean = 4.83: SD = 0.75), as well as high levels of understanding from week 0 (Mean = 5.06; SD = 0.78) to week 8 (Mean = 5.31: SD = 0.48) (See Figure 2). Furthermore, the feasibility scores did not change from week 0 (Mean = 4.28: SD = 0.89) to week 8 (Mean = 4.22: SD = 0.71), indicating that the intervention was feasible for the 8-week period.

The *acceptability* scores deviated from the ML teachers’ qualitative explanations of encountering challenges in time for preparation of the intervention. The scores for *understanding* of ML could indicate a potential pathway for teachers starting with a theoretical understanding of the intervention, which then expanded into a practical understanding through the implementation strategies (e.g., the courses), qualifying the teachers to interpret the intervention and apply it in practice:

“*I found the second part (of the courses at the school) to be helpful… to hear the introduction to this mindset of Move & Learn, with the attached elements. I cannot remember it by heart, but I have the didactic tool and principles on my desk, and it is a way of thinking that I need to incorporate in my teaching*”

(School 4, Teacher 1)

The *feasibility* of the intervention was rated slightly lower than the acceptability and understanding, and this rating remained unchanged after 8 weeks in the quantitative data. Connecting this to the qualitative ML findings, the experience of the pedagogical development of teaching can serve as a feasibility factor. Hence, personal affirmation allowed the teachers to maintain the motivation to carry on:

“*So, it has been great to be challenged to think about it in a new way and to be confirmed that, well, I can actually do it when encountering another topic. It would normally be easier just to follow the pupils’ book*”

(School 6, Teacher 2)

For another teacher, pupils’ reactions became a self-reinforcing cycle, which made the intervention feasible:

“*As soon as I start, they know what it is about. Then it just runs smoothly. It has been really inspiring*”

(School 7, Teacher 2)

The overall thematic reflexive analysis of the qualitative data for the ML intervention had three core themes: *instant perception, translation of intervention, and context matters* (see the full theme summary table in Supplementary Materials Section V).

*Instant perception* describes the existence of fast and frequent changes in the class environment, which affected the prerequisites for implementing ML at the individual level, thus affecting the teachers’ acceptance and ability to make sense of the ML intervention. Teachers expressed that they were initially motivated to engage, but, for example, sick leave among colleagues or chaos in the classroom could instantly change this perspective.

Setbacks because of restless and disturbing pupils or the instability of staff power affected the project’s feasibility day to day.

The theme *translation of intervention* was grounded in the way teachers interpreted the intervention. This interpretation was formed primarily by the staff capacity strategies (see Table 1), such as educational courses and materials used to support the implementation of the intervention. Moreover, this interpretation developed from a hesitant to a reflected attitude towards the way the interventions could be applied over the 8 weeks.

The theme *context matters* covered, for example, the support by leaders or colleagues and the school as a system. Further, *content matters* covered recipient reactions and feedback.

### 3.2. Appropriateness

The appropriateness of an intervention is a determinant contributing to the likelihood of proper implementation. This was measured by post-intervention questions about appropriateness at various levels, e.g., the school level and teacher level.

#### 3.2.1. Run, Jump & Fun

In the post-intervention survey, teachers implementing the RJF intervention rated the appropriateness of the intervention in relation to (1) school strategy (mean = 4.26), the learning environment in the class (mean = 4.40), and their own practices (mean = 4.00), respectively. Since the RJF intervention took time away from curricular activities, some of the teachers in the project felt that it compromised learning activities. It was rated as less appropriate for the teachers’ own practices than for the learning environment in the class, indicating that it was more appropriate for the pupil’s needs compared to the teachers’ needs in teaching practice.

In the qualitative analysis for RJF, *appropriateness* constituted a component in the overarching theme of *boarding to start building*. The dilemma of teachers spending time on RJF at the expense of time for subjects teaching time, is illustrated in this quote:

“*So, I think it has worked well for me. However, I would have liked to have been able to better integrate it into the subjects*”

(School 1, Teacher 3)

#### 3.2.2. Move & Learn

The results for teachers implementing the ML intervention in the post-intervention survey showed how appropriate they found the intervention in relation to school strategy (M = 4.92), the learning environment in the class (M = 4.64), and their own practices (M = 5.42), respectively. In the interviews, an ML teacher amplified the following:

“*It was a way for me to remind myself that it is also important to include movement when teaching 3rd grade.—That it is important to let them move around and get their bodies moving as well*”

(School 6, Teacher 2)

In general, teachers perceived the ML intervention as being very appropriate.

### 3.3. Implementation

The implementation was measured by the outcomes of *adoption* and *fidelity,* which were self-reported in the weekly SMS survey and the post-intervention survey and were covered qualitatively in the interviews.

#### 3.3.1. Run, Jump & Fun

**Figure 3:**
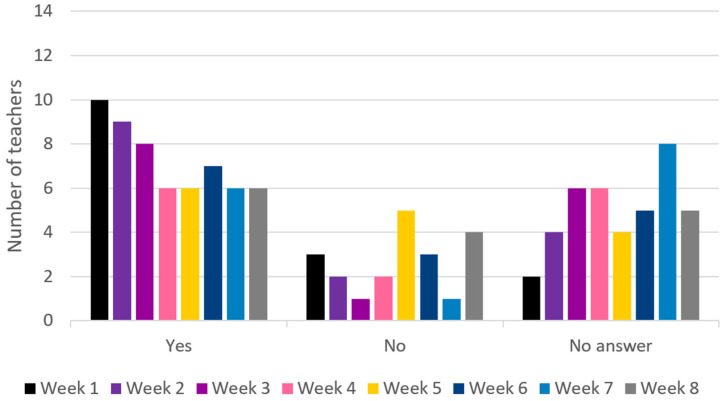
The adoption of RJF by teachers in weeks 1–8. Adoption: Did you implement RJF sessions this week?

*Adoption* refers to teachers’ decisions to take action and implement the intended ideas behind the intervention. The RJF teachers rated *adoption* high with M = 4.62 (range 3–6) in the post-intervention survey. The number of completed sessions is illustrated in Figure 3. The number of teachers who adopted RJF was relatively consistent, with a tendency for more teachers to report adoption in the first weeks. However, caution should be taken when interpreting the data as an average of 5 out of 14 teachers did not answer between week 2 and week 8.

**Figure 4:**
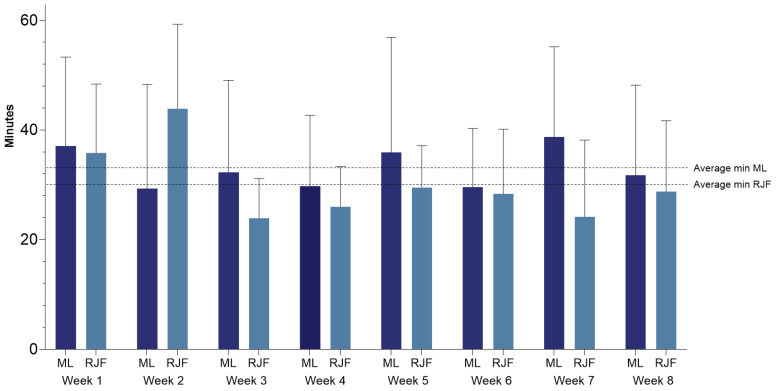
Average sessions’ duration for every week in minutes for ML and RJF.

*Fidelity* refers to the degree to which the intervention is delivered as intended. This measure is divided into fidelity to intervention instructions and fidelity to dose. Fidelity also includes adherence to the principles. Fidelity to intervention instructions (courses, materials, and intervention principles) was rated M = 4.54 (range 2–6) by RJF teachers (*n* = 13). *Fidelity* to dose was quantitatively measured with teacher self-reports each week. During the 8 weeks, the average minutes pr. session of RJF was 28 min. The weekly results are illustrated in Figure 4 and show consistent fidelity to the target of 30 min.

The qualitative data related mainly to the overarching theme of *boarding to start building* and demonstrated how strategies for *adoption* can affect the attitude towards the interventions positively.

“*It was not really about creating some advanced games and activities with all sorts of equipment; it was actually just about making it fun and involving to as many pupils as possible. They should be activated, raise their heart rate, and feel that they were a part of a community*”

(School 5, Teacher 4)

The fidelity findings from the interviews demonstrated that the teachers experienced the target of 30 min four out of five days a week differently. For example, the RJF teachers report that activities with a higher degree of self-determination could be sustained for extended periods without requiring significant effort from the teacher. One RJF teacher reflected on the fact that music motivated the pupils to keep moving for longer without teacher instructions:

“*So, we found some different music with power and in that way, we could easily go on for half an hour with the children*”

(School 5, Teacher 1)

Several teachers mentioned that adhering to the principles enabled them to implement the intervention consistently and with high quality. *Fidelity* to principles was, therefore, a critical factor in the success of the implementation.

#### 3.3.2. Move & Learn

**Figure 5:**
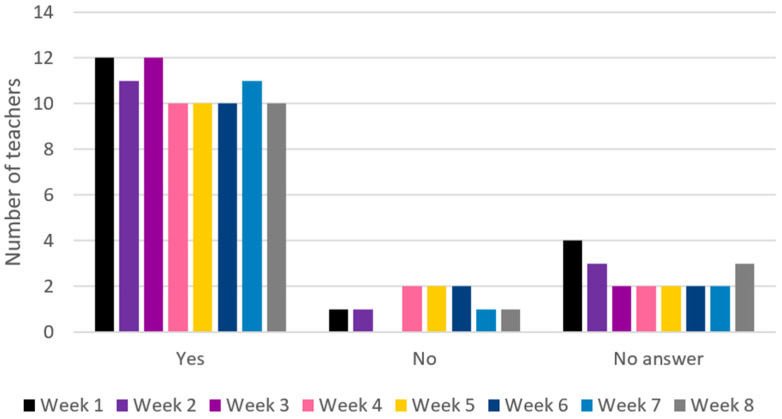
The adoption of ML by teachers in weeks 1–8. Adoption: Did you implement ML sessions this week?

The ML teachers rated *adoption* high at M = 5.25 (range 4–6) in the post-intervention survey. The results for ML adoption measured in the weekly SMS survey are shown in Figure 5 and illustrate that the teachers report having delivered ML sessions consistently throughout the 8-week period. Only a few teachers did not answer or reported not having applied any ML activities. This aligns well with the adoption score in the post-intervention survey.

Accordingly, in the post-intervention survey, the ML teachers (*n* = 12) rated their fidelity to the intervention instructions (courses, materials, and intervention principles) as M = 4.33 (range 2–5). Fidelity to duration was assessed through the teacher’s self-report. The average for each session of ML was 33 min (see Figure 4).

The qualitative ML data relate mainly to the overarching theme of *translation of intervention*. Although the intervention strategies positively affected the teachers and promoted the adoption of the intervention, in some cases, the students who were recipients affected the adoption negatively:

“*What you [ACTIVE SCHOOL] are providing is both inspiring and the materials are comprehensive and excellent. However, it has been challenging for me to implement it with 3rd grade*”

(School 7, Teacher 1)

Another interesting aspect was that teachers felt empowered with the intervention tools to refine and construct their own embodied learning activities. This led to self-sufficiency, concurrently enhancing fidelity to ML like this teacher points out:

“*…I have, to a large extent, received tons of good ideas and have been able to work on them myself. I have also been given tools for it, like how we think it ourselves, without having it served to us. It has been so fantastic…much better than I dared to hope for*”

(School 7, Teacher 3)

## 4. Discussion

The aim of this 8-week feasibility study was to explore the outcomes of the implementation of the two teacher led interventions “Run, Jump & Fun” and “Move & Learn”, targeting third grade students in seven schools. RJF can be conceptualized as extra non-curricular MVPA implemented within the school day while ML is conceptualized as embodied learning integrated into curricular time. Based on the measurement and evaluation of the feasibility and implementation, the study indicates promising results for both interventions, separately. However, the study highlights points related to the general acceptability of the interventions and identifies the necessity for refinements to the specific implementation strategies for both interventions. These conclusions are based on specific qualitative findings from interviews with teachers from both the RJF and ML interventions.

This early identification of challenges is vital for future research and larger studies in physically active learning, embodied learning and in congenial school-based PA. There is a need of feasibility studies, to ensure that larger-scale school studies are tailored to the context and practice of the teachers [50]. Accordingly, in the following section, our results regarding the antecedent assessments of *understanding*, *acceptability*, and *appropriateness* will be discussed, followed by the actual implementation outcomes, including the adoption of the interventions and the fidelity to the interventions’ instructions. At the end, there will be an outline of recommendations, and how these impact the development of future school-based PA interventions.

### 4.1. Antecedent Assessments

Antecedent assessments of implementation can work as a preliminary evaluation of the actual implementation and may predict if an intervention will be adopted and implemented with fidelity [44].

#### 4.1.1. Understanding

The teacher’s understanding of the interventions was primarily built through the courses, with 62% of the ML teachers and 85% of the RJF teachers rating the courses as one of the most important strategies. The general need for further teacher education has been addressed in a range of previous studies, and was summarized in a recent meta-synthesis which addressed physically active learning (PAL) teacher training [51]. This meta-synthesis identified sub-themes like “Tailored ongoing support” and “PAL examples, demonstrations and direct experiences”, which resemble the themes used in the present study. In ACTIVE SCHOOL, the courses consisted of an introduction to the theory of EL and the principles behind the interventions, hands-on practice with the materials provided, and the delivery of the activities in the specific facilities of the single school. The results from the present study highlighted a need for more demonstrations and practical experiences with the activities in the courses instead of a large amount of theory, which has likewise been found in other school-based PA studies [52].

#### 4.1.2. Acceptability

The quantitative data from the RJF intervention showed a high level of acceptability. However, some teachers emphasized that they would like to make adaptations to RJF to create task-relevant activities that are integrated into the subjects’ matter, while keeping the intensity high. Such a perspective could resonate with previous research, indicating that teachers mainly focus on activities that contribute to pupils’ learning [53]. In addition, this study indicates that some teachers found it difficult to comply with the RJF principles, thus encouraging the creation of self-defined pedagogical goals such as collaboration or participation.

Some teachers considered ML to be time demanding to prepare. An extended intervention period might have given the teachers more positive experiences with the embodied learning activities, and as these activities become a routine, the teachers might spend less time on preparation. This was found in a Norwegian study investigating the effect of daily physical activity on children’s academic performance [54]. After two years, the researchers performed a qualitative follow-up study and found that previous positive experiences influenced how teachers adopted PAL. Also the aforementioned meta-synthesis concludes that teacher training can reduce the initial burden and time constraints of preparation [51]. To create lasting change, it is recommended that teachers become experienced in designing their own activities. Additionally, the long-term nature of an intervention like ML, which involves personal and professional development, should be clearly communicated and recognized. The findings from this study indicated that teachers’ motivation for sustaining the intervention was based in the fact that they perceived PAL as relevant to their role as teachers.

Some teachers in both interventions highlighted that the time allocation for the project was not prioritized by the school management. This is a barrier documented in other studies [55,56]. A review by Nathan et al. (2018), identified the domain of “environmental context and resources”. This domain impacted the implementation of PA in schools and included among other factors the priority of time for PA. The review addresses the complex financing models in schools, which can be a reason for not prioritizing the time teachers need to prepare and implement PA in schools [56].

It is essential to allocate time for teachers to adapt their practices to emerging new theories. This time investment is crucial for professional growth and should be prioritized when implementing theory-based interventions.

#### 4.1.3. Appropriateness

The RJF teachers rated the intervention’s appropriate for their own practice to some degree. One explanation for this may be the experienced challenge of taking time away from academic lessons to implement MVPA activities with no task relevance. However, substantial evidence has demonstrated that taking time away from academic lessons and substituting it with PA does not compromise learning outcomes [57,58,59,60].

The ML teachers assessed the intervention as being appropriate to a high or very high degree in relation to their own practice and in relation to the strategy of the schools. This aligns with results from a survey across Danish schools in 2022, where 64% of the school personnel responded that PA implemented as learning activities within subjects was a prioritized strategy at their school [61].

### 4.2. Implementation Outcomes

#### 4.2.1. Adoption

The actual implementation outcomes of the study are the *adoption* and *fidelity* of the interventions [44]. The process of RJF establishing started by building the teachers’ attitudes towards the intervention and implementing shared decision-making on how to adopt the intervention at each school. In other studies, this has been identified as a key factor for adoption [62,63]. However, it is rare that PA intervention studies have such an establishment process as an implementation strategy, though it is recommendable to have it to facilitate ownership of the interventions before starting the actual implementation period.

Conversely, many studies have included courses and intervention-specific artifacts as adoption strategies. For example, in the ASK study, a custom developed website with videos and content for PAL lessons was available, and the teachers were provided with supportive PAL equipment [64]. Another study provided teachers with a “resource kit” containing activity cards and programs, and the material was demonstrated in practice in teacher training sessions [65]. In our study, teachers were provided with written material, video material, and relevant website links with inspirational teaching activities.

A qualitative meta-synthesis by Daly-Smith et al. (2021) covers other factors affecting adoption. For example, the benefits of the pupils’ motivation and the gradual development of the teachers’ mindset facilitate adoption. Simple steps when building up experiences are necessary for the adoption of the intervention. Furthermore, fostering a positive and motivated attitude among the teacher group is important [51]. In ACTIVE SCHOOL, these elements were emphasized in the introduction course. The courses had an appreciative approach, where the teachers’ prior experiences of implementing PA or PAL functioned as a point of departure for further development. Thereby, previous teacher experiences were acknowledged as being valuable and important. Within the courses, teachers and educators co-created teaching activities with ML and RJF, respectively. This may enable the development of new values related to PAL and motivate teachers to engage with students in a new collaborative way [53], ultimately strengthening adoption.

#### 4.2.2. Fidelity

Fidelity can be measured with several criteria [66]. In this study, the criteria were focused on dose delivered in minutes, adherence to the principles, and the quality of the delivered interventions. It has been emphasized that the fidelity of interventions with educational aspects is a complex phenomenon that is difficult to measure [67], and the findings from this study are consistent with this notion. The report of the dose, in minutes and days delivered, was relatively stable, but the results must be interpreted with caution, since teachers tend to exaggerate when self-reporting [68].

The teacher-driven delivery, which is seen in many school-based PA studies [37], could have affected fidelity, because the teachers’ diversity of competences affects their comprehension of the intervention instructions. However, the understanding and hands-on use of the intervention principles might have facilitated the distinction between and critical choice of relevant intervention teaching activities and may have led to adherence to the intended intervention.

Adherence to the ML embodied learning intervention was explored qualitatively, to cover the aforementioned complexity. The fact that teachers found it difficult to reach the target of 30 min of embodied learning activities, and sometimes only planned one activity usually lasting less than 30 min, is important knowledge about real-life issues. For ML, adherence to the target number of minutes may not necessarily be of the highest priority. Rather, adherence needs to be supported by principles advocating for the activities, which foster cognitive engagement influenced by factors other than time and intensity. For example, interactions with other students or achieving bodily experiences with subject matter. Therefore, adherence to the principles concerning the quality of embodied learning might be the most important criterion in measuring fidelity to the ML intervention.

### 4.3. Recommendations and Implications

The overall findings from this feasibility study demonstrate that the two interventions are feasible for schools to implement. Furthermore, the study revealed numerous findings, which were discussed above, and these are synthesized as the recommendations below. These will inform the refinement of the interventions before a larger ACTIVE SCHOOL RCT study is conducted.

The recommendations outlined below are structured to address each of the various implementation strategies experienced in a step-wise manner to promote comprehensive adoption, from the initial establishment meetings with schools to sustaining activities after the end of an intervention. They are relevant to research on school-based interventions in general, and can be points of attention when conducting larger studies. These recommendations may also give valuable insight into schools planning to implement PA.

Recommendations:

1. Shared decision-making and organizing the local intervention model specific to each school’s individual context can positively impact implementation [69,70].
2. The personnel required to implement an intervention must be defined in the early stages and the workforce in schools must be taken into account [71]. Intervention providers must clearly define and inform school leaders about the time required in teachers’ yearly time allocation, without compromising other mandatory tasks. Importantly, an agreement on the total amount of time allocated for the project should be formalized in a contract.
3. Courses to foster adoption must ideally be provided in several sessions, particularly when the intervention covers a complete school year, in order to support teachers’ gradual development in implementing a PA intervention [72]. Practical engagement and demonstrations must be highly prioritized over theory.
4. Courses must present teaching materials in a clear and exemplary manner to inspire teachers to use them [65]. Materials must come in varied formats, such as videos, booklets, posters, and a single, easily navigable webpage, and should encompass diverse intervention deliverers’ preferences [64].
5. For school-based PAL or embodied learning interventions, it is important to communicate the long-term perspective of the interventions. Planning and re-thinking teaching for embodied learning is a step-by-step process and ongoing practice leads to self-confidence and routine [54,73]. Further, for these types of interventions, attention to a specific intensity can be replaced by attention to a movement-centered approach, where the intervention entails learning activities with the body which are widely integrated into the learning process [73].
6. To avoid mixing when there is more than one intervention type in the same study, there must be a clear distinction between these interventions in all communications, especially in exemplary course activities.

These recommendations may serve as a foundation when designing or expanding school-based PA interventions. It is worth noting that the recommendations build on the rigorous study of teacher practice. The synthesis of these recommendations is an attempt to bridge the gap between evidence and implementation to create relevant and implementable interventions [74]. This gap has recently addressed in the literature in a position paper from 2024, highlighting the need to rethink the role of schools as settings for PA promotion [40]. Bengoechea et al. (2024) highlight the need to take a system approach towards school-based interventions. Further, when scaling-up perspectives, the position paper states that research should draw on practice-based evidence to achieve the most meaningful knowledge to tailor complex school interventions.

This mixed-methods feasibility study attempts to bridge the gap mentioned earlier by addressing points relevant to the preparation of any PA intervention in a primary school. The recommendations represent findings from the complex context and are relevant across diverse school-based interventions, both for interventions that incorporate embodied learning and MVPA sessions into the school day and for PE interventions, for instance.

Although ACTIVE SCHOOL is not a full program with a whole school system theory behind it [75], it contains elements, like implementation strategies, which are defined as bridging in relation to the practice–implementation gap [76]. Future school-based studies with PA interventions could bridge this gap even better. Interventions should be developed in cooperation with both teachers and students [77]. This would increase the potential of school interventions to also possibly affect the PA behavior and habits of children at home and during leisure time [78].

### 4.4. Strengths and Limitations

The use of mixed methods to assess feasibility should be considered a strength of this study. Thus, the quantitative data show the degree of feasibility, and the qualitative data explain why and how the implementation is feasible. Applying the overall implementation theory from the CFIR OAD has further strengthened the study. This study is, to our knowledge, one of the first studies to use the CFIR OAD to guide which implementation outcomes should be included, and how to relate these to the exploration.

Other strengths of the ACTIVE SCHOOL study are the co-design of the interventions with teachers (development phase) and the delivery of the intervention by teachers (this feasibility study and the RCT study). These approaches have enabled more context-specific and feasible interventions to the very complex setting of schools [28,69], which may foster sustainability over a longer period [37].

In general, the results from this feasibility study must be interpreted with caution due to the limited sample size both in regard to schools and teachers involved as informants, which limits the reliability and generalizability of the findings. However, this feasibility study has informed the future randomized controlled ACTIVE SCHOOL study and can be informative for other researchers aiming to develop feasible physical activity interventions in schools. Furthermore, while the study only had seven schools enrolled, the schools were diverse regarding their size, geography, and experience with PA implementation. The main outcomes were subjectively reported measures of adoption of the intervention and adherence in minutes. Additional objective measures could have strengthened the study, e.g., a more detailed evaluation of the quality of embodied learning in the ML intervention (e.g., observations of teaching) and a more precise measure of the amount/intensity of the PA in the RJF intervention (e.g., accelerometer measures).

Finally, since the study assessed the feasibility of the interventions and tested a preliminary series of tests for a future RCT study [79], the schools experienced unintended stress and overload during the 8-week period regarding communicative duties, academic test sessions with children, test days with the research team, participation in courses, and implementation of the interventions. This would probably be perceived as less stressful if the duration of the study had been longer and the project activities had been conducted over several months.

## 5. Conclusions

The present feasibility study showed that both the embodied learning intervention (ML) and the intervention with additional MVPA sessions during school time (RJF) were acceptable, adoptable, and feasible for teachers and their pupils, in accordance with our hypothesis. However, the qualitative data results led to recommendations for further tailoring to the specific school context and minor adjustments of the two interventions when expanding to a larger sample size in a study with a longer duration. The most notable adjustment recommended was to add an establishment process to both interventions. The establishment process proved to be a sensible and helpful way to address the complexity of the school setting and allowed each school to go through a decision-making process to promote adoption. Further, school-based PA or embodied learning should align with the educational goals of the specific school system to be appropriate for implementation and increase sustainability. The experiences gained from the study emphasize the importance of continuing to rigorously develop and test the feasibility of different theory-based approaches to school-based PA interventions, acknowledging the complexity of the school context.

## Figures and Tables

**Table 1:** Methods used to measure implementation determinants and outcomes.

Source	Content	Measurement
Usage Rating Profile Intervention online survey	A 29-item self-report electronic questionnaire covering six indicators of adaptability and implementation of interventions. Five out of six subscales were included: (1) Acceptability, (2) Understanding, (3) Feasibility, (4) System Climate and (5) System Support.	A 6-point scale ranging from 1 = strongly disagree to 6 = strongly agree. They were measured at week 0 before the pilot period, after the introduction course, and at week 8 (the end of the intervention).
**Teacher post-intervention online survey**	Custom quantitative (ratings) regarding adoption, feasibility, adoption, implementation and sustainability: For example: To which degree have you adopted the intervention? To what degree did the implementation strategies aid you? Qualitative (open-ended) questions, for example: Why were these strategies the most important to you?	A 6-point scale ranging from 1 = not at all to 6 = to a very high degree. Boxes in the survey where respondents could write further explanations for their quantitative ratings.
**A weekly short survey sent out by SMS to the smartphone**	Custom quantitative (ratings) regarding adoption and adherence to the interventions.	Adoption: yes/no Adherence to intervention principles on a 6-point scale ranging from 1 = not at all to 6 = to a very high degree. Delivery of minutes each day—open the box to write the number of minutes delivered.
**Semi-structured post-intervention group interviews** **(present)**	Interview questions (examples): Implementation: What did it take to implement this intervention? Adaptation: How has your practice changed throughout the eight weeks?	Interviews were recorded and transcribed.
**Observations**	Field observations of teachers delivering the interventions.	Jotted field notes

**Table 2:** Intervention characteristics and implementation strategies for Move & Learn and Run, Jump & Fun.

Intervention	Level of Strategy	Strategy	Keywords
Run, Jump & Fun	Staff delivery	4 × 30 minutes weekly with MVPA intensity	Applying seven principles of RJF intervention
	Staff capacity (understanding)	Two courses (6 and 3 hours)	Introduction to principles, practice tools and implementation Planning of activities Tailored feedback
	Staff capacity (understanding)	Teaching materials	Posters, booklets, lesson plans and links with specific activities and videos
	System climate (school support)	Run, Jump & Fun establishing process.	3 Meetings before and one during the intervention. Meeting guidance provided by ACTIVE SCHOOL
	System climate (school support)	Team meetings	For teachers and school principals. Meeting guidance provided by ACTIVE SCHOOL.
Move & Lean	Staff delivery	2 × 30 minutes of ML Math weekly 2 × 30 minutes of ML Danish weekly	Applying five principles of ML intervention
	Staff capacity (understanding)	Two courses (6 and 3 hours)	Introduction to principles, practice tools and implementation Planning of activities Tailored feedback
	Staff capacity (understanding)	Teaching materials	Posters, booklets, lesson plans and links with specific activities and videos
	System climate (school support)	Team meetings	For teachers and school principals. Meeting guidance provided by ACTIVE SCHOOL

## Data Availability

Data not available due to restrictions (data is in Danish language).

## Supplementary Materials

The following supporting information can be downloaded at: https://www.mdpi.com/article/10.3390/ijerph22010067/s1, Material I: Principles of ‘Run, Jump & Fun’ and ‘Move & Learn’ interventions; Material II: Terminology nexus of Consolidated Framework for Implementation Research Outcomes Addendum (CFIR OAD) and Usage Rating Profile—Intervention (URP-I); Material III: Usage Rating Profile—Intervention survey ACTIVE SCHOOL version; Material IV: Interview guide for group post-interviews; Material V: Themes and Sample Quotes from Move & Learn group interviews and Run, Jump & Fun group interviews.

## Abbreviations

CFIR OAD	Consolidated Framework for Implementation Research Outcomes Addendum Diagram
ML	‘Move & Learn’ intervention
MVPA	Moderate to vigorous Physical activity
PA	Physical activity
PAL	Physically Active Learning
PE	Physical Education
RCT	Randomized Controlled Trial
RJF	‘Run, Jump & Fun’ intervention
SMS	Short Message Service
URP-I	Usage Rating Profile-Intervention
