# Supplementary material for: Implementation Outcomes and Recommendations of Two Physical Activity Interventions: Results from the Danish ACTIVE SCHOOL Feasibility Study
Source: Int J Environ Res Public Health. 2025 Jan 7;22(1):67. doi: 10.3390/ijerph22010067 (PMC11765277; doi:10.3390/ijerph22010067)
Supplement: Supplementary file 1 [file ijerph-22-00067-s001.zip › ijerph-3345858-supplementary.pdf]

## Supplementary Material

**Material I**

**Principles of 'Run, Jump & Fun' and 'Move & Learn' interventions**

| Run, Jump & Fun (RJF) principles | Move & Learn (ML) principles |
|---|---|
| • Connect a pedagogical aim to the RJF activity.<br>• Pay attention to the target group and prepare activities in advance.<br>• Instructions must be short and precise.<br>• Modify the activities, so inactive breaks do not occur.<br>• Be aware of the duration and intensity of activities.<br>• Foster the possibility of autonomy in activities.<br>• Discover opportunities for activities in alternative settings. | • Integrate curriculum-based PA in two Danish and two Math lessons per week.<br>• The bodily engagement must underpin the curricular aim of the lesson.<br>• The body can and must be a part of the immersion or the solution of an academic activity during the intervention time.<br>• Organize group activities with a maximum of three members in each group.<br>• Foster the possibility of autonomy in activities. |

**Material II**

**Terminology nexus of Consolidated Framework for Implementation Research Outcomes Addendum (CFIR OAD) and Usage Rating Profile - Intervention (URP-I).**

| Outcome terminology<br>**CFIR Outcomes Addendum Diagram** | Outcome terminology<br>**Usage Rating Profile - Intervention** |
|---|---|
| Acceptability | Acceptability, Understanding |
| Feasibility | Feasibility, Understanding |
| Appropriateness | System Climate |
| Implementation Climate | System Support |
| Implementation (Adoption and fidelity) | |

| Sustainability | |
|---|---|

**Material III**

**Usage Rating Profile – Intervention survey ACTIVE SCHOOL version**

| No (origin-nal) | Statement | Factor | Strongly disagree 1 | Disagree 2 | Slightly disagree 3 | Slightly agree 4 | Agree 5 | Strongly agree 6 |
|---|---|---|---|---|---|---|---|---|
| 1 | This intervention is an effective choice for addressing a variety of problems | ACCEPTABILITY | 1 | 2 | 3 | 4 | 5 | 6 |
| 2 | I would need additional resources to carry out this intervention. | SYSTEM-SUPPORT | 1 | 2 | 3 | 4 | 5 | 6 |
| 3 | I would be able to allocate my time to implement this intervention. | FEASIBILITY | 1 | 2 | 3 | 4 | 5 | 6 |
| 4 | I understand how to use this intervention. | UNDERSTANDING | 1 | 2 | 3 | 4 | 5 | 6 |
| 6 | I am knowledgeable about the intervention procedures. (duration, dose, principles) | UNDERSTANDING | 1 | 2 | 3 | 4 | 5 | 6 |
| 7 | The intervention is a fair way to handle the child's behavior problem. | ACCEPTABILITY | 1 | 2 | 3 | 4 | 5 | 6 |
| 8 | The total time required to implement the intervention procedures would be manageable. | FEASIBILITY | 1 | 2 | 3 | 4 | 5 | 6 |
| 9 | I would not be interested in implementing this intervention. | ACCEPTABILITY reverse code these items when scoring | 1 | 2 | 3 | 4 | 5 | 6 |
| 10 | My administrator would be supportive of my use of this intervention. | SYSTEM-CLIMATE | 1 | 2 | 3 | 4 | 5 | 6 |
| 11 | I would have positive attitudes about implementing this intervention. | ACCEPTABILITY | 1 | 2 | 3 | 4 | 5 | 6 |
| 12 | This intervention is a good way to handle the child's behavior problem. | ACCEPTABILITY | 1 | 2 | 3 | 4 | 5 | 6 |
| 13 | Preparation of materials needed for this intervention would be minimal. (teaching exercises and materials for students) | FEASIBILITY | 1 | 2 | 3 | 4 | 5 | 6 |

| | | | | | | | | |
|---|---|---|---|---|---|---|---|---|
| 14 | Use of this intervention would be consistent with the mission of my school. | SYSTEM-CLIMATE | 1 | 2 | 3 | 4 | 5 | 6 |
| 16 | Implementation of this intervention is well matched to what is expected in my job. | SYSTEM-CLIMATE | 1 | 2 | 3 | 4 | 5 | 6 |
| 17 | Material resources needed for this intervention are reasonable | FEASIBILITY | 1 | 2 | 3 | 4 | 5 | 6 |
| 18 | I would implement this intervention with a good deal of enthusiasm | ACCEPTABILITY | 1 | 2 | 3 | 4 | 5 | 6 |
| 19 | This intervention is too complex to carry out accurately. | FEASIBILITY reverse code these items when scoring | 1 | 2 | 3 | 4 | 5 | 6 |
| 20 | These intervention procedures are consistent with the way things are done in my system | SYSTEM-CLIMATE | 1 | 2 | 3 | 4 | 5 | 6 |
| 21 | This intervention would not be disruptive to other students. | ACCEPTABILITY | 1 | 2 | 3 | 4 | 5 | 6 |
| 22 | I would be committed to carrying out this intervention. | ACCEPTABILITY | 1 | 2 | 3 | 4 | 5 | 6 |
| 23 | The intervention procedures (principles) easily fit i with my current practices. | ACCEPTABILITY | 1 | 2 | 3 | 4 | 5 | 6 |
| 24 | I would need consultative support to implement this intervention | SYSTEM-SUPPORT | 1 | 2 | 3 | 4 | 5 | 6 |
| 25 | I understand the procedures (principles) of this intervention. | UNDERSTANDING | 1 | 2 | 3 | 4 | 5 | 6 |
| 26 | My work environment is conducive to implementation of an intervention like this one. | SYSTEM-CLIMATE | 1 | 2 | 3 | 4 | 5 | 6 |
| 29 | I would require additional professional development in order to implement this intervention. | SYSTEM-SUPPORT | 1 | 2 | 3 | 4 | 5 | 6 |

Note: The Home-school factor was excluded (statement 5,15,28) and also 27.

**Material IV**
**Interview guide for group post-interviews**

| Theme | Questions | Aim |
|---|---|---|
| **Introduction** | Welcome and presentation of the aim of the interview.<br><br>Short status of the project. | To include the interview in the overall overview. |

| | | |
|---|---|---|
| | Framing of the interview.<br><br>Rules for respondents<br>Respondents present themselves. | Create the framework for the social space.<br><br>To get the respondents involved and manage their voices in relation to the recording. |
| **Part 1:**<br><br>**Acceptability** | • How were you involved in the decision to have Active School here at the school?<br><br>• Was it clear to you what the interventions were about?<br><br>• How did the initial meetings, course, and materials/principles sent to you, work in terms of preparing you for the project?<br><br>• How has the attitude and support for the project been at the school? - From colleagues, management, and parents? | To focus on context, management, colleagues and attitudes towards the intervention. |
| **Part 2:**<br>**Feasibility**<br>**Implementation Climate** | • How have you experienced receiving various initiatives from Active School? (meetings, courses, extra materials, visits from external experts, Movement Mentors course?)<br><br>• How have the initiatives from Active School affected your work on the project?<br><br>• What has it required of you to do this? - How has your motivation been?<br><br>• How would you assess your performance in the effort?<br><br>• How well has the effort matched the way you teach/work as an educator? - And for the school you work at?<br><br>• How have your students reacted?<br><br>• How has the management acted?<br><br>• How satisfied are you with your participation? | To explore how the teachers have implemented the interventions and which attributes they found necessary for this. |
| **Del 3:**<br>**Adoption**<br>**Implementation** | • Have you been able to maintain the dose, duration and principles of the intervention or have you adjusted something to better suit you/class?<br><br>• Is there something you have NOT done at all? (what about the meetings, for example?) | To discover how teachers delivered the intervention and which adaptations they made. |

| | | |
|---|---|---|
| | • Has your way of working with the intervention changed during the 8 weeks?<br><br>• If you were to participate for an entire year, what should the timeline look like? How much support and education is needed to maintain your practice? | |
| **Part 4:**<br><br>(not related to the feasibility study)<br>**The didactical models for ML** | • How have you used the model and how have they potentially guided your process?<br><br>• Does it work as a didactic tool or as a model or something else? What do you think of it? | To explore didactics position in their teacher life and how movement and the body become part of their didactics with the model. |
| **Appreciation of their participation** | Thanks a lot for you and your pupils' participation in the project. | |

**Material V**

**Themes and Sample Quotes from Move & Learn group interviews**

| Theme: **Instant perception** | | |
|---|---|---|
| Codes | CFIR OAD construct | Sample quotations |
| Teacher buy-in | Acceptability<br>Implementation readiness | *School 7, teacher 4: "This is really something. That path, we need to open our eyes to."* |
| Teacher DNA | Appropriateness<br>Acceptability | *School 4, teacher 3:: "I think it's really awesome, because that's how I remember. When you've been on the ground yourself. I'm the type of person who gets tired when I sit still. So when I'm involved myself, that's when things are remembered."*<br><br>*School 7, teacher 3: "I'm not the sporty type. And I'm not the one who goes out and plays. That's just not me. So in that way, it's been far from it. And yet I've been inspired."* |
| Teacher journey and sensemaking | Acceptability<br>Feasibility | *School 6, teacher 2: "So it's been cool to be challenged to think about it a new way, so you're also affirmed in, well, I can actually do that, also when you come across other topics that would normally be easier to just follow the book."*<br><br>*School 4, teacher 2: "It should be something that becomes integrated, which is difficult and challenging at first. But I would say especially after meeting with [the course educator], where I got some background knowledge on this, I think it was better... we need to make it a part of our thinking process... getting it fully integrated, that takes some time."* |

| | | |
|---|---|---|
| Why participate | Appropriateness<br>Implementation readiness | *School 6, teacher 2: "The project is good for just keeping a handle on the fact that I'm actually obligated to do it, for some other people, not just myself and the pupils. Even though you're already good at it, it's always nice to have an extra factor that sort of comes into play.".* |
| Theme: **Translation of intervention** | | |
| Codes | CFIR OAD construct | Sample quotations |
| Interpretation of the intervention | Feasibility<br>Adoption<br>Implementation | *School 6, teacher 2: "… we also sit and praise ourselves and say that we've been good at it and we use movement and such. But maybe we've been better at making some energizers different kinds that maybe didn't necessarily directly relate to the subject, and there I have, paid attention to also incorporating the subject into it."*<br><br>*School 4, teacher 1: "So because there I got the opportunity to delve into this I realized that I always had the idea that movement should be about getting the heart rate up, but it didn't have to be."*<br><br>*School 2, teacher 2: "You become more aware of... whether the activity actually has something to do with what we want or if it's just an activity for the sake of being an activity."* |
| Teacher specific Strategies | Appropriateness<br>Feasibility<br>Adoption | *School 4, teacher 1: "It helped after we had that team meeting where the educator was there, where we simply got the peace for this. And I think it's just really really important to have it in the team meetings."*<br><br>*School 7, teacher 4: "Well, I don't think I've ever been to such inspiring courses. (Another teacher acknowledges and says: Yes, agree). I'm a little impressed by what the instructor he gives us and the way he gives it to us."* |
| Foster Self-determination | Appropriateness<br>Adoption | *Skole 4, L1: Så på den måde kan man sige, der er jeg blevet rykket lidt i det der. Kunne jeg gå ind og gøre noget, så børnene også føler at de bestemmer lidt?*<br><br>*School 4, teacher 1: "So in that way, you could say, I've been pyushed a bit in that direction. Could I do something, so the children also feel like they have a say?"* |
| Theme: **Context matters** | | |
| Codes | CFIR OAD Construct | Sample quotations |
| System and individual support | Implementation<br>Climate<br>Appropriateness<br>Feasibility (feasible for context) | *School 4:*<br>*Teacher 4 (a pedagogue): "I really wished I could have been there [to the course]."*<br>*Teacher 3: "And it wasn't actually prioritized by the management for the whole team to be allowed to attend."* |

| | | |
|---|---|---|
| | "Features of context" | *Teacher 3: "So we didn't have pedagogues at the course, but it could have been really great."*<br><br>*School 6:*<br>*Teacher 1: "Yes, and we mentioned it at the parent meeting... there were many positive responses."*<br>*Teacher 2: "So there has been support from management and we are also going to tell about it at a staff meeting for all."*<br>*Teacher 1: "We have five focus areas, one of which is movement in teaching and movement in general, so we want to focus on that."*<br><br>*School 7, L1: "When the two of us sat together in math for the short time we had [the educator] last, you can also just see how quickly we can come up with ideas."* |
| Recipient outcomes | Acceptability<br>Feasibility | *School 2, teacher 1: "When we did ML I experienced sometimes that everyone was working, and none was off the guard."*<br><br>*School 7, teacher 4: "It's also a formative process for the students, having to learn to be in this movement. And sometimes there's just chaos. At least in 3.a. Especially when we're outside of their usual seats and routines."* |

**Themes and Sample Quotes from Run, Jump & Fun group interviews**

| Theme: **Boarding to start building** | | |
|---|---|---|
| Codes | CFIR construct | Sample quotations |
| Staff buy-in and DNA | Acceptability | *School 1, teacher 2: "It was something that already aligned with my own practice. So it was something I wanted to participate in.* |
| Grasping the intervention | Appropriateness and understanding -> Adoption | *School 1, teacher 1: "I would say, your materials, the fact that you don't have to go out and invest a lot in equipment. That's very smart. It means that everyone can participate in everything."* |
| Context support systems | System climate | *School 5, teacher 3: "If we've been lacking fly swatters or large dice or cones or something like that, they've quickly stepped in. So in that way, we've felt support from our fellow teachers. But the management, it's like they've forgotten..."*<br><br>*School 2, teacher 1: "I actually think he (department head) has been quite involved because he has attended several meetings, and he has essentially supported us. He has given us free hands to do what we wanted... But sometimes I have been unsure about what his role should be in it, other than supporting us."* |

| Theme: **Hands-on engagement matters** | | |
|---|---|---|
| Codes | CFIR construct | Sample quotations |
| Staff sensemaking and journey | Understanding -> Adoption | *School 1, teacher 3:"I learn it through playing, and I think it sticks better that way. And I... in a way, can familiarize with these games and make them my own."*<br><br>*School 3, teacher 2: "It was nice to try it out on my own body, how it actually should feel. Because I think, you get some associations with the words that are being used. When I hear "high pulse", I think, it's something where you run a marathon…but it didn't have to be that way. It could also be a bit more moderate"*<br><br>*School 3, teacher 2: " I feel that I have developed it in terms of trying something out and adjusting along the way but also by talking with you along the way."* |
| Practice implementation | Feasibility | *Skole 2, teacher 2: "It required a lot of planning I feel... we did that also during our team meetings, but then I just felt that it affected and took time away from other important things."*<br><br>*School 5, L1: "And it was super easy to transfer to a substitute, if we weren't there one day. It was just to write to the substitute; you just have to do Run, jump & fun, just ask the kids what it is."* |
| Principles affecting students and sustainment | Feasibility<br><br>Sustainability | *School 3, teacher 2: "I was a bit unsure at first, what does high pulse for half an hour actually mean, what does it entail exactly?"*<br><br>*School 5, teacher 3: "Well, we went through them [the principles] already at the course on the first day, where we started talking about them being some guidelines. "I think that they (the principles) are quite self-evident when planning a lesson. All that is listed there is something a teacher does"* |
